# Associated Factors for Quality of Life, Anxiety, and Depression in Patients with Chronic Heart Failure and Their Family Caregivers: A Cross-Sectional Study in Japan

**DOI:** 10.1089/pmr.2024.0056

**Published:** 2024-12-24

**Authors:** Mayumi Niitani, Hiroyuki Sawatari, Yuri Takei, Naoko Yamashita

**Affiliations:** ^1^ Department of Nursing and Health Science, Graduate School of Medicine, Ehime University, Ehime, Japan.; ^2^ Department of Perioperative and Critical Care Management, Graduate School of Biomedical and Health Sciences, Hiroshima University, Hiroshima, Japan.

**Keywords:** chronic heart failure, family caregiver, quality of life, anxiety, depression

## Abstract

**Background:**

Family caregivers (FCs) need to provide regular assistance and good quality care to patients to prevent the deterioration of chronic heart failure (CHF); therefore, they may have physical and mental distress. However, physical and mental distress in FCs of patients with CHF in Japan is unclear.

**Objective:**

This study aimed to clarify the quality of life (QoL), anxiety/depression, and associated factors in patients with CHF and their FCs.

**Design:**

We conducted a multicenter cross-sectional survey using a questionnaire between 2016 and 2017 among patients with CHF and their FCs. Demographic data were extracted from medical records. Health-related QoL and anxiety/depression were assessed using the Short Form-12 and the Hospital Anxiety and Depression Scales, respectively.

**Results:**

Of 286 patients and FCs (response rate 57.2%), the physical component summary and mental component summary (MCS) scores of FC were higher than those of patients (*p* < 0.001 and *p* = 0.047, respectively). The incidence of anxiety and depression in patients with CHF was 7.0% and 10.8%, respectively, whereas that in FC was 10.1% and 12.6%. In multivariable analysis, the MCS score of FC was associated with the MCS score of patients (β = 0.22, *p* < 0.001). Anxiety in FC was associated with anxiety (β = 0.30, *p* < 0.001) in patients, respectively.

**Conclusions:**

It is necessary to carefully monitor the physical and mental condition of patients with CHF and provide palliative care in collaboration with the palliative care team as needed. Stabilizing the patient’s physical and mental condition through palliative care may also help alleviate the suffering of FC.

## Introduction

Globally, the prevalence of chronic heart failure (CHF) and its associated loss in health have been constantly increasing over the past several decades,^1^ and with an aging population, the number of patients with CHF is expected to increase. In Japan, where the population is aging rapidly, the number of patients with heart failure (HF) is estimated to reach 1.3 million by 2030.^2^ In the management of HF, preventing rehospitalization due to acute exacerbations is important to prolong healthy life expectancy and improve quality of life (QoL). The Japanese Cardiac Registry of Heart Failure in Cardiology showed that the common causes of readmission due to HF exacerbation were inadequate salt or water restrictions, overwork, inadequate medication, and mental or physical stress. It has also been clarified that HF is exacerbated by some diseases, such as infectious diseases, arrhythmias, hypertension, and diabetes.^3^ The actual situation in patients with CHF suggests that supporting self-management would be important to prevent readmission and death in these patients.^4–6^

A previous study showed that approximately 75% of patients with CHF lived with their families,^7^ and the presence of family caregivers (FCs) was important for self-management at home in these patients. A guideline of the Japanese Circulation Society for the treatment of CHF also states to “strive to improve the self-care of patients through education and consultation support for patients and their families.”^8^ Caregivers, including family members, often need to help patients with self-management.

Therefore, the burden on FCs who support patients with unstable physical and mental conditions would be high. It has been reported that the frequency of anxiety and depression in patients with CHF is high, estimated at 11–45% and 10–60%, respectively.^9^ Furthermore, excessive intake of water and salt, as well as infectious diseases, such as respiratory infection, could be significant factors for causing decompensated HF. FCs are required to pay high attention and constantly monitor patients with CHF to manage mental distress and underlying diseases of patients, which might lead to decreased mental health of the caregivers.

In Europe and the United States, attention from medical staff has been paid to the distress of families caring for patients with HF since the early stages of HF.^10,11^ A study of spouses of patients with HF showed that anxiety and depression were higher in spouses than in patients, and the psychological state of spouses was affected by the burden of HF in the patient’s life.^12^ A survey of 213 spouses of patients undergoing cardiac rehabilitation reported that 66% of spouses had mental distress.^13^ Family members who were caring for the patients with both CHF and depressive symptoms showed significantly higher levels of caregiving burden and lower QoL, compared with those caring for patients with CHF without depressive symptoms.^14^ Although FCs are often regarded as the resource that supports patients, they should also be supported by medical staff, according to research in Europe and the United States.^15–18^

FCs are recognized in the National Consensus Statement as well as by other national and international palliative care professional organizations as part of the unit of care that is the primary focus of palliative care clinical practice and research.^19–20^ However, the physical and psychological states of Japanese FCs of patients with CHF are rarely accounted for. Thus, in this study, we examined QoL, anxiety, and depression in FCs, as well as in patients with CHF.

### Objective

This study aimed to clarify the QoL, psychological states (i.e., depression and anxiety), awareness of self-care behaviors, and their influencing factors in patients with CHF and their FCs.

## Methods

### Study design and participants

A multicenter, cross-sectional survey was conducted from April 2016 to March 2017, using a semi-structured questionnaire. Consent to participate in the study was obtained from FCs of patients by completing the questionnaire. This survey was conducted with the approval of the Hiroshima University Epidemiological Research Ethics Review Board and Research Cooperation Hospital (# E306 Ethics Committee for Epidemiology of Hiroshima University).

The participants in this study were patients diagnosed with CHF who were followed up at four hospitals in Hiroshima Prefecture and their families who were recognized as the main supporters. Patients with CHF were excluded if they (1) were aged <20 years, (2) were under treatment for cancer, or (3) had dementia or cognitive dysfunction. If the FCs accompanied patients at the outpatient clinic, they were also asked to participate in the study. If the FCs did not attend, patients were asked to take home this study participation request form and the questionnaire for FCs.

### Measurements

Clinical information was collected immediately after enrollment. Demographic data, including age, sex, occupation, educational history, and relationship with the patient, were self-reported. Clinical characteristics of the patients with CHF, including underlying disease, number of months since the first hospitalization due to the causative disease, left ventricular ejection fraction (LVEF), plasma *N*-terminal pro-brain natriuretic peptide, and long-term care insurance service usage status, were extracted from the medical records.

### Quality of life

The QoL of patients with CHF and their FCs was assessed using the validated Japanese version of the Medical Outcome Study Short Form-12, version 2 (SF-12v2).^21,22^ The SF-12v2 consists of physical and emotional well-being subscales, namely, the physical component summary (PCS) and the mental component summary (MCS), respectively. The total score on each subscale ranges from 0 to 100 points, with higher scores indicating better physical and emotional well-being.^21^ The reliability and validity have been well documented in caregivers and patients with CHF.^23,24^

### Anxiety and depression

Anxiety and depression were assessed using the validated Japanese version of the Hospital Anxiety and Depression Scale (HADS),^25,26^ with individuals with a score of 11 points or higher being rated as anxious and depressed. The HADS comprises seven questions relevant to either generalized anxiety or depression, with each item containing a 4-point (0–3) response category. The possible scores for anxiety and depression range from 0 to 21 points. Anxiety and depression were scored separately. A score of 0–7, 8–10, and 11–21 points for either subscale was regarded as being in the normal range, a borderline case, and an abnormal case, respectively. We defined individuals as having anxiety and/or depression when the score was higher than 10 points.

### Self-care behavior

The Japanese version of the European Heart Failure Self-Care Behavior Scale (EHFScBS) Ver.2^27,28^ was used to evaluate self-care behavior. The EHFScBS is a 12-item self-administered questionnaire that covers items concerning the self-care behavior of patients with CHF, such as daily weighing, fluid restriction, and contacting health care providers about gaining weight.

### Data analysis

The data for analysis were presented as median (interquartile range [IQR]), number (%), and standardized beta (β). Mann-Whitney U test was used to compare between the two groups after the assessment of Gaussian distribution using the Kolmogorov-Smirnov test for continuous variables and the chi-squared test for nominal or binary variables. For the general linear model, we first conducted a univariate analysis. Variables with a *p* value <0.10 in the univariate analysis were included in the multivariate analysis (multiple regression analysis). Non-Gaussian distributed variables were converted to logarithms to obtain a Gaussian distribution for the general linear model. Statistical significance was set at *p* < 0.05. All the statistical analyses were performed using SPSS version 23 (IBM Corp., Armonk, NY, USA).

## Results

**FIG. 1.**
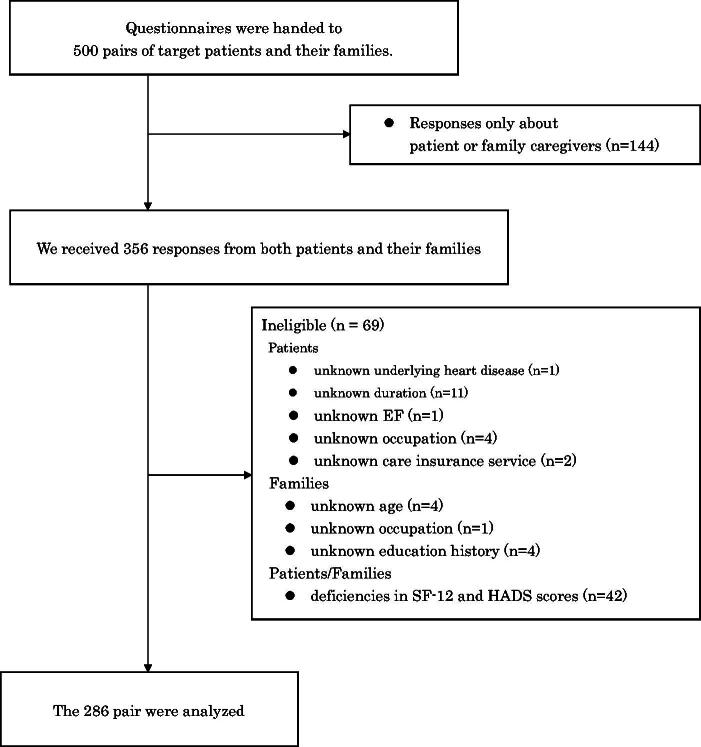
Respondent flowchart.

During the study period, 538 patients were asked to participate, and 500 of them who consented to participate received the questionnaire. Of the 500 questionnaires distributed, 356 responses (71.2%) were received from patients and/or their families. We excluded 70 responses where it was reported that the survey was filled out only by the patient or by the FC and finally analyzed 286 responses (response rate 57.2%) that were answered by both the patients with CHF and their FCs (Fig. 1). The FCs of patients with CHF were their husbands (12.9%), wives (57.7%), children (21.7%), parents (3.5%), siblings (1.4%), or others (2.7%), of which 230 (80.1%) lived with the patients.

Table 1 shows the characteristics of the patients and their FCs. The median (IQR) age of the patients was 72.0 (65.0–79.0) years, with most being men (204 [71.3%]), 52 patients (18.2%) were receiving social support. The patients were with a median (IQR) LVEF of 55.0% (43.7–64.0%) and had been living with their HF diagnosis for a median (IQR) of 65.0 (24.0–125.0) months. Seventy-nine patients (27.6%) were employed and having received high-school education or less (180 [64.5%]). The causes of HF were ischemic heart disease in 126 (44.1%), cardiomyopathy in 73 (25.5%), hypertensive heart disease in 42 (14.7%), arrhythmia in 26 (9.1%), congenital heart disease in 11 (3.8%), and valvular disease in 3 (1.0%) patients.

**Table 1.** Characteristics of the Patients with Chronic Heart Failure and Their Family Caregivers

	Patients	Family caregivers	*p*-Value
Number	286	286	—
Age, y.o.	72.0 (65.0–79.0)	66.0 (57.0–74.0)	<0.001
Male, *N* (%)	204 (71.3)	63 (22.0)	<0.001
Social support, *N* (%)	52 (18.2)	—	—
LVEF, %	55.0 (43.7–64.0)	—	—
NT-proBNP, pg/mL (*N* = 250)	255.0 (69.0–916.0)	—	—
Past time after the diagnosis, months	65.0 (24.0–125.0)	—	—
Employment, *N* (%)			
Employed	79 (27.6)	126 (44.1)	<0.001
Education, *N* (%)			
Less than or equal to high school	180 (64.5)	164 (57.7)	
Junior college	24 (8.6)	70 (24.6)	
Greater than or equal to college	75 (26.9)	50 (17.6)	<0.001
Underlying heart diseases, *N* (%)			
Ischemic heart diseases	126 (44.1)	—	—
Cardiomyopathy	73 (25.5)	—	—
Hypertensive heart diseases	42 (14.7)	—	—
Arrhythmia	26 (9.1)	—	—
Congenital heart diseases	11 (3.8)	—	—
Valvular diseases	3 (1.0)	—	—
Others	5 (1.7)	—	—
SF-12, points			
PCS	43.5 (36.8–50.3)	49.1 (42.8–53.9)	<0.001
MCS	48.8 (40.1–55.3)	49.9 (42.1–57.2)	0.047
HADS			
Anxiety score, points	4.0 (2.0–7.0)	5.0 (2.0–8.0)	0.85
Depression score, points	6.0 (3.0–9.0)	5.5 (3.0–9.0)	0.94
Presence of anxiety, *N* (%)	20 (7.0)	31 (10.1)	0.11
Presence of depression, *N* (%)	31 (10.8)	37 (12.6)	0.44
EHFScBS			
Self-care score, points	35.0 (28.0–40.0)	—	—

Values are shown as median (25 percentile–75 percentile) or number (percent).

EHFScBS, European Heart Failure Self-Care Behavior Scale; HADS, Hospital Anxiety and Depression Scale; LVEF, left ventricular ejection fraction; MCS, mental component summary; *N*, number; NT-proBNP, *N*-terminal pro-brain natriuretic peptide; PCS, physical component summary; SF-12, Short Form-12; y.o., years old.

Regarding the FCs, the median (IQR) age was 66.0 (57.0–74.0) years, with most being women (223 [78.0%]), 126 (44.1%) were employed and having received high-school education or less (164 [57.7%]).

About the QoL assessed using the SF-12, the median PCS was 43.5 (36.8–50.3) points for patients and 49.1 (42.8–53.9) points for FCs, with family scores being significantly higher (*p* < 0.001). The median MCS was 48.8 (40.1–55.3) points for patients and 49.9 (42.1–57.2) points for FCs, with no significant difference (*p* = 0.047).

Regarding the psychological state, the median value of anxiety and depression scores, assessed by the HADS, of patients with CHF were 4.0 (2.0–7.0) points and 6.0 (3.0–9.0) points, respectively. There were no significant differences between patients with CHF and FCs in the anxiety and depression scores (FC: 5.0 [2.0–8.0] points and 5.5 [3.0–9.0] points, respectively). Anxiety and depression occurred in 20 (7.0%) and 31 (10.8%) patients with CHF, respectively, as well as in 31 (10.1%) and 37 (12.6%) FCs, respectively (*p* = 0.11 and *p* = 0.44, respectively).

The median self-care scale was 35.0 (28.0–40.0) points.

### Factors associated with the QoL of FCs

FCs were divided into two groups, with high and low median values of physical and mental QoL assessed using the SF-12, and the factors associated with each subscale of QoL were assessed (Table 2).

**Table 2.** Clinical Characteristics Stratified by the Median Values of PCS-F and MCS-F

	PCS-F			MCS-F		
	Higher	Lower	*p*-Value	Higher	Lower	*p*-Value
Number	138	137	—	138	137	—
Family caregivers						
Age, y.o.	61.5 (52.0–68.0)	71.0 (63.0–78.0)	<0.001	67.0 (58.0–74.0)	65.0 (57.0–73.0)	0.42
Male, *N* (%)	34 (24.6)	25 (19.0)	0.31	32 (23.2)	28 (20.4)	0.66
Employment, *N* (%)						
Employed	79 (57.2)	44 (32.1)	<0.001	60 (43.5)	63 (46.0)	0.72
Education, *N* (%)						
Less than or equal to high school	67 (48.6)	89 (65.9)		88 (64.2)	68 (50.0)	
Junior college	39 (28.3)	29 (21.5)		30 (21.9)	38 (27.9)	
Greater than or equal to college	32 (23.2)	17 (12.6)	0.01	19 (13.9)	30 (22.1)	0.051
Patients						
Age, y.o.	69.0 (64.0–76.0)	74.0 (67.0–82.0)	0.001	71.0 (65.0–78.0)	72.0 (66.0–80.0)	0.28
Male, *N* (%)	96 (69.6)	100 (73.0)	0.59	98 (71.0)	98 (71.5)	1.00
Social support, *N* (%)	14 (10.1)	35 (25.5)	0.001	13 (9.4)	36 (26.3)	<0.001
LVEF, %	55.6 (44.0–63.8)	54.0 (45.0–64.0)	0.75	53.7 (45.0–64.0)	55.0 (44.0–64.0)	0.81
NT-proBNP, pg/mL	181.0 (46.0–681.0)	321.4 (111.0–1236.0)	0.01	226.3 (59.5–728.0)	330.0 (75.4–1002.8)	0.16
Past time after the diagnosis, months	59.0 (21.0–115.0)	68.0 (27.0–131.0)	0.18	55.0 (21.0–124.0)	80.0 (27.0–127.0)	0.19
Underlying heart diseases, *N* (%)						
Ischemic heart diseases	65 (47.1)	57 (41.6)		69 (50.0)	53 (38.7)	
Cardiomyopathy	32 (23.2)	36 (26.3)		27 (19.6)	41 (29.9)	
Hypertensive heart diseases	21 (15.2)	21 (15.3)		21 (15.2)	21 (15.3)	
Arrhythmia	13 (9.4)	11 (8.0)		8 (5.8)	16 (11.7)	
Congenital heart diseases	5 (3.6)	6 (4.4)		8 (5.8)	3 (2.2)	
Valvular diseases	0 (0.0)	3 (2.2)		1 (0.7)	2 (1.5)	
Others	2 (1.4)	3 (2.2)	0.65	4 (2.9)	1 (0.7)	0.06
Employment, *N* (%)						
Employed	44 (31.9)	32 (23.4)	0.14	39 (28.3)	37 (27.0)	0.89
Education, *N* (%)						
Less than or equal to high school	80 (58.8)	93 (70.5)		89 (66.4)	84 (62.7)	
Junior college	11 (8.1)	11 (8.3)		9 (6.7)	13 (9.7)	
Greater than or equal to college	45 (33.1)	28 (21.2)	0.09	36 (27.6)	37 (27.6)	0.64
SF-12, points						
PCS	45.3 (37.5–52.5)	42.4 (35.8–48.9)	0.07	44.2 ± 8.6	41.6 ± 10.5	0.03
MCS	50.5 (42.0–56.8)	46.8 (39.1–53.5)	0.07	45.3 (38.0–51.5)	51.4 (43.7–57.6)	<0.001
HADS, points						
Anxiety	4.0 (2.0–7.0)	4.0 (2.0–8.0)	0.37	5.0 (3.0–8.0)	4.0 (2.0–6.0)	0.004
Depression	5.0 (3.0–8.0)	6.0 (4.0–9.0)	0.20	7.0 (4.0–10.0)	5.0 (3.0–7.0)	<0.001
EHFScBS, points						
Self-care score	36.5 (28.0–41.0)	34.0 (27.0–39.0)	0.053	35.0 (28.0–40.0)	36.0 (28.0–40.0)	0.30

Values are shown as median (25 percentile–75 percentile), mean ± SD or number (percent).

MCS-F, mental component summary in family caregiver; PCS-F, physical component summary in family caregiver.

FCs’ PCS scores (PCS-F) were higher in those who were younger (61.5 vs. 71.0, *p* < 0.001) and more likely to be employed (57.2% vs. 32.1%, *p* < 0.001). On the contrary, the MCS scores of FCs (MCS-F) were higher in those with fewer social support users (9.4% vs. 26.3%, *p* < 0.001), lower MCS scores of patients (MCS-P) (45.3 vs. 51.4, *p* < 0.001), and higher depression scores of patients (7.0 vs. 5.0, *p* < 0.001).

The results of the multivariate analysis are shown in Table 3: no factors were significantly associated with the PCS-F score (Table 3A); for the MCS-F, there was a significant association with the FC education (β = −0.24, *p* < 0.001) and with the MCS-P score (β = 0.22, *p* < 0.001) (Table 3B).

**Table 3.** Relationships Between the Family Caregivers’ Quality of Life and the Patients’ Quality of Life

	Univariate		Multivariate	
	Β	*p*-Value	***β***	*p*-Value
(A) PCS-F				
Family caregivers				
Log age	−0.36	<0.001	−0.25	0.003
Male	0.05	0.42	—	—
Employed	0.31	<0.001	0.17	0.03
Education	0.22	<0.001	0.06	0.42
Patients				
Log age	−0.18	0.003	0.10	0.22
Male	−0.07	0.24	—	—
Social support	−0.12	0.053	−0.01	0.92
Log LVEF	0.03	0.62	—	—
Log NT-proBNP	−0.13	0.046	−0.09	0.20
Log past time after the diagnosis	−0.06	0.32	—	—
Employed	0.15	0.01	0.03	0.69
Education	0.21	0.001	0.17	0.02
SF-12				
Log PCS	0.16	0.01	0.11	0.14
Log MCS	0.07	0.27	—	—
EHFScBS				
Log self-care score	0.12	0.045	0.03	0.71
(B) MCS-F				
Family caregivers				
Log age	0.04	0.53	—	—
Male	0.02	0.70	—	—
Employed	−0.01	0.87	—	—
Education	−0.25	<0.001	−0.24	<0.001
Patients				
Log age	−0.01	0.87	—	—
Male	0.01	0.89	—	—
Social support	−0.20	0.001	−0.04	0.57
Log LVEF	0.10	0.09	0.09	0.14
Log NT-proBNP	−0.05	0.46	—	—
Log past time after the diagnosis	−0.07	0.23	—	—
Employed	0.03	0.67	—	—
Education	−0.09	0.14	—	—
SF-12				
Log PCS	0.18	0.004	0.13	0.06
Log MCS	0.26	<0.001	0.22	<0.001
EHFScBS				
Log self-care score	0.08	0.21	—	—

### Factors associated with anxiety and depression in FCs

FCs were also divided into two groups, with high and low median value of anxiety and depression scores assessed using the HADS. As shown in Table 4, higher anxiety in FCs was significantly associated with the MCS-P score (45.4 vs. 51.3, *p* < 0.001), anxiety (6.0 vs. 3.0, *p* < 0.001), and depression (7.0 vs. 5.0, *p* < 0.001) in patients. Furthermore, higher depression scores in FCs were significantly associated with the MCS-P score (46.7 vs. 50.9, *p* < 0.001) and depression (7.0 vs. 5.0, *p* < 0.001) in patients.

**Table 4.** Clinical Characteristics Stratified by the Median Values of Anxiety and Depression

	Anxiety in the family caregivers			Depression in the family caregivers		
	Higher	Lower	*p*-Value	Higher	Lower	*p*-Value
Number	139	133	—	136	136	
Family caregivers						
Age, y.o.	66.0 (57.0–74.0)	66.0 (56.0–73.0)	0.55	65.5 (57.0–74.0)	66.0 (57.0–74.0)	0.92
Male, *N* (%)	28 (20.1)	32 (24.1)	0.47	33 (24.3)	27 (19.9)	0.47
Employment, *N* (%)						
Employed	60 (43.2)	64 (48.1)	0.47	60 (44.1)	64 (47.1)	0.72
Education, *N* (%)						
Less than or equal to high school	74 (53.6)	80 (60.2)		74 (54.8)	80 (58.8)	
Junior college	34 (24.6)	34 (25.6)		32 (23.7)	36 (26.5)	
Greater than or equal to college	19 (21.7)	19 (14.3)	0.27	29 (21.5)	20 (14.7)	0.35
Patients						
Age, y.o.	73.0 (65.5–80.0)	71.0 (65.0–78.0)	0.52	73.0 (65.0–80.0)	71.0 (65.0–78.0)	0.21
Male, *N* (%)	100 (71.9)	93 (69.9)	0.79	94 (69.1)	99 (72.8)	0.59
Social support, *N* (%)	23 (23.7)	17 (12.8)	0.03	33 (24.3)	17 (12.5)	0.02
LVEF, %	54.0 (43.2–64.0)	55.1 (43.7–63.8)	0.69	55.6 (42.1–64.6)	53.0 (44.0–63.0)	0.47
NT-proBNP, pg/mL	329.0 (94.3–991.0)	240.8 (49.1–863.0)	0.15	321.4 (102.0–1087.0)	236.0 (57.2–729.0)	0.08
Past time after the diagnosis, months	72.0 (27.0–124.0)	59.0 (22.0–124.0)	0.38	73.0 (29.5–126.0)	59.5 (21.0–124.0)	0.38
Underlying heart diseases, *N* (%)						
Ischemic heart diseases	54 (38.8)	63 (47.4)		50 (36.8)	67 (49.3)	
Cardiomyopathy	38 (27.3)	32 (24.1)		42 (30.9)	28 (20.6)	
Hypertensive heart diseases	23 (16.5)	17 (12.8)		20 (14.7)	20 (14.7)	
Arrhythmia	15 (10.8)	11 (83)		12 (8.8)	14 (10.3)	
Congenital heart diseases	5 (3.6)	6 (4.5)		7 (5.1)	4 (2.9)	
Valvular diseases	2 (1.4)	1 (0.8)		2 (1.5)	1 (0.7)	
Others	2 (1.4)	3 (2.3)	0.78	3 (2.2)	2 (1.5)	0.34
Employment, *N* (%)						
Employed	35 (25.2)	41 (30.8)	0.35	31 (22.8)	45 (33.1)	0.08
Education, *N* (%)						
Less than or equal to high school	87 (64.0)	86 (66.2)		84 (63.6)	89 (63.6)	
Junior college	13 (9.6)	8 (6.2)		15 (11.4)	6 (4.5)	
Greater than or equal to college	36 (26.5)	36 (27.7)	0.59	33 (25.0)	39 (29.1)	0.11
SF-12, points						
PCS	41.6 ± 10.3	44.7 ± 8.7	0.01	41.9 ± 10.6	44.5 ± 8.4	0.03
MCS	45.4 (38.7–51.9)	51.3 (43.7–57.3)	<0.001	46.7 (38.3–52.5)	50.9 (42.7–57.3)	<0.001
HADS, points						
Anxiety	6.0 (3.0–9.0)	3.0 (1.0–6.0)	<0.001	5.0 (3.0–8.0)	3.0 (1.0–7.0)	0.001
Depression	7.0 (4.0–10.0)	5.0 (3.0–8.0)	<0.001	7.0 (4.0–10.0)	5.0 (3.0–7.0)	<0.001
EHFScBS						
Self-care score, points	33.0 (25.5–39.0)	37.5 (30.0–42.0)	0.001	35.0 (28.0–40.0)	36.0 (28.0–41.5)	0.37

Values are shown as median (25 percentile–75 percentile), mean ± SD or number (percent).

Subsequent multivariate analysis revealed that anxiety in FCs was related to anxiety in patients (β = 0.30, *p* < 0.001) (Table 5A). However, no factor was significantly associated with depression in FCs (Table 5B).

**Table 5.** Relationships Between the Family Caregivers’ Hospital Anxiety and Depression Scale Score and the Patients’ Hospital Anxiety and Depression Scale Score

	Univariate		Multivariate	
	***β***	*p*-Value	***β***	*p*-Value
(A) Anxiety in the family caregivers				
Family caregivers				
Log age	−0.04	0.51	—	—
Male	−0.02	0.81	—	—
Employed	0.02	0.78	—	—
Education	0.13	0.03	0.11	0.06
Patients				
Log age	−0.004	0.95	—	—
Male	−0.04	0.47	—	—
Social support	0.16	0.009	0.06	0.30
Log LVEF	−0.09	0.15	—	—
Log NT-proBNP	0.08	0.25	—	—
Log past time after the diagnosis	0.04	0.51	—	—
Employed	−0.08	0.19	—	—
Education	0.04	0.51	—	—
HADS				
Log anxiety	0.41	<0.001	0.30	<0.001
Log depression	0.34	<0.001	0.12	0.11
EHFScBS				
Log self-care score	−0.21	<0.001	−0.17	0.005
(B) Depression in the family caregivers				
Family caregivers				
Log age	−0.04	0.54	—	—
Male	0.01	0.87	—	—
Employed	−0.03	0.62	—	—
Education	0.12	0.049	0.10	0.15
Patients				
Log age	0.01	0.84	—	—
Male	−0.05	0.45	—	—
Social support	0.13	0.03	0.01	0.88
Log LVEF	−0.05	0.38	—	—
Log NT-proBNP	0.11	0.09	0.01	0.89
Log past time after the diagnosis	−0.03	0.57	—	—
Employed	−0.12	0.055	−0.08	0.25
Education	0.00	1.00	—	—
HADS				
Log anxiety	0.26	<0.001	0.10	0.26
Log depression	0.34	<0.001	0.27	0.002
EHFScBS				
Log self-care score	−0.14	0.03	−0.14	0.048

## Discussion

To the best of our knowledge, this is the first multicenter cross-sectional study to demonstrate the relationship between QoL, anxiety, and depression among FCs and patient characteristics in Japan. This study showed that FCs’ psychological QoL and anxiety were associated with patients’ QoL (*p* < 0.001) and anxiety (*p* < 0.001).

This study showed that the QoL of FCs, assessed using the SF-12, was higher than that of the patients, which is consistent with the findings from a previous study conducted by Dellafiore et al.^29^ Dellafiore et al. showed that the mean ± standard deviations of PCS-P and MCS-P scores were 35.32 ± 9.52 and 44.87 ± 10.10 points, respectively, whereas that of PCS-F and MCS-F scores were 48.76 ± 8.31 and 48.69 ± 9.20 points, respectively, indicating that the QoL of patients was lower than that of FCs. Furthermore, the national standard values of PCS and MCS scores of healthy individuals in their 70s were 44.0 ± 9.6 and 55.9 ± 8.0 points, respectively^30^; the PCS-P score in this study was slightly lower and the PCS-F score was higher. The reason for this is that many patients were in their 70s, whereas the FCs were younger (median age, 66.0 years), which may have caused a difference in the evaluation of physical function. In addition, the fact that the PCS-P score of patients with CHF was similar to that of healthy individuals might be partly due to the fact that the severity of CHF was not high. The PCS-P score in this study was higher than that reported by Dellafiore et al.^29^ However, the median values of the MCS score in patients and FCs in this study were lower than the national standard value for individuals in their 70s. It is clear that the mental QoL of both patients and their FCs decreased, even though the physical QoL remained the same, compared with that of healthy individuals. Furthermore, it was revealed that the MCS-F score was influenced by the FC’s educational history (β = −0.24, *p* < 0.001) and MCS-P score (β = 0.22, *p* < 0.001). Honjo et al.^31^ showed that low educational attainment was associated with worse mental health in Japan. FCs who have experienced a patient’s ICU admission report multidimensional wellness and stress, and appropriate interventions are needed at the time of discharge.^32^ Interventions that take into account the educational history of FCs and reduce the burden of support at home are needed.

In this study, the proportion of patients with depression was lower than that in previous studies.^9^ Although HF severity was unknown as noted in the limitations, anxiety and depression occurred in 20 (7.0%) and 31 (10.8%) patients with CHF, respectively, as well as in 31 (10.1%) and 37 (12.6%) FCs in this study. In addition, the depression scores of patients, as well as the depression and anxiety scores of FCs, were higher than the average scores in a survey of 1234 Japanese cancer survivors assessed using the HADS.^33^ As the severity of the disease increases, the incidence of depression also increases, and the comorbidity of depression worsens HF,^34^ and so, it is important to screen for depression and anxiety at the early stage of onset, as recommended in the United Kingdom and the United States.^35,36^ Furthermore, the results of multivariate analysis in this study showed that anxiety in patients with CHF was significantly associated with anxiety in their FCs. The Integrated Palliative care Outcome Scale (IPOS) is a scale used to assess the palliative care needs of patients with HF.^37^ Matsunuma et al.^38^ used the IPOS to evaluate 101 patients, with 36% of patients classified as NYHA II, 30% as III, and 3% as IV. The results of the study showed that the physical symptoms that the patients reported as interfering with their lives were dyspnea (29%) and drowsiness (29%), and the psychological symptoms experienced were family anxiety (28%), patient anxiety (25%), and depression (18%). For the patients, the anxiety felt by their families is also a source of mental distress, and it was found that family anxiety is partly caused by a lack of information. Although communication with health care providers is important to improve the lack of information, patients report that they find it difficult to say or do what really bothers them, and health care providers also find it difficult to get to know their patients and discuss their perspectives.^39^ Only cardiology specialists often have difficulty alleviating the suffering of patients with HF and their FCs, so collaboration with a palliative care team is recommended when necessary.

### Limitations

There were several limitations in this study. First, there was a lack of data by NYHA or ACCF/AHA stage on the severity of HF. This made it difficult to compare the introduction of palliative care with previous studies. Hamatani et al.^40^ reported that among NYHA IV participants, the mean age was 70 ± 15 years, 78% were male, the median LVEF was 24% (16–45), and the median BNP was 1180 pg/mL (616–2734). Similarly, Kawaguchi et al.^41^ reported a mean age of 73.5 years, 65% male, median LVEF of 25% (15–40), and median BNP of 927 pg/mL (584–1834). Compared with the results of their study, it is assumed that the participants in this study were patients with a less severe condition than NYHA IV. Our cohort was not that advanced, and these patients traditionally have minimum symptom burden; nevertheless, it is noteworthy that the FCs had low psychological QoL and suffered from anxiety.

The second limitation of this study is that only a total of 268 pairs of patients and FCs participated in this study. Hiroshima Prefecture, where this survey was conducted, has a standardized death rate from HF of 110.085,^42^ ranking 12th in Japan, which is rather high considering the risk of death due to HF. In addition, the four hospitals that recruited the participants are facilities that play a central role in cardiovascular medicine in Hiroshima Prefecture, and the purpose of this study was appropriately clarified with the participants.

## Conclusions

In conclusion, this study showed that there is an association between psychological QoL and anxiety in patients with CHF and their FCs, regardless of the severity of HF. Their QoL and anxiety may be related to lack of symptom control or limited information about prognosis or overall condition. It is recommended to monitor palliative care needs from an early stage and collaborate with the palliative care team when necessary.

## Abbreviations Used

AHA: American Heart Association
ACCF: American College of Cardiology Foundation
NYHA: New York Heart Association
